# Design of global climate control based on fuzzy systems with concept of carbon emissions

**DOI:** 10.1371/journal.pone.0333846

**Published:** 2025-10-14

**Authors:** Saira Naseer, Wer-Jer Chang, Muhammad Shamrooz Aslam, Muhammad Hashim Bukhari, Hazrat Bilal, Muhammad Javid Nawaz

**Affiliations:** 1 School of Economics and Management, Quanzhou University of Information Engineering, Quanzhou, Fujian, People’s Republic of China; 2 Department of Marine Engineering, National Taiwan Ocean University (NTOU), Keelung, Taiwan; 3 School of Computer Science and Technology/School of Artificial Intelligence, China University of Mining and Technology, Xuzhou, People’s Republic of China; 4 Saudi AramcoStar Building LIP, Dhahran, Saudi Arabia; 5 School of Information Science and Technology, University of Science and Technology of China, Hefei, Anhui, People’s Republic of China; 6 School of Economics and Management, Nanjing University of Science and Technology, Nanjing, People’s Republic of China; Agricultural Sciences and Natural Resources University of Khuzestan, IRAN, ISLAMIC REPUBLIC OF

## Abstract

The global carbon–climate system is a highly complex and dynamic network characterized by multiple feedback loops between interconnected components. Addressing the risks of climate change requires active intervention across these components (Atmospheric level, Surface ocean, and Terrestrial biosphere). Consequently, this research introduces a new mathematical fuzzy control theory to explore how control mechanisms, incorporating both open and closed–loop, can help guide the carbon–climate system toward more stable and sustainable levels. **First**, a fuzzy mathematical generalization as a compartmental dynamical model is proposed for a formal analysis of closed–loop control strategies for climate regulation. **Second**, the challenge of managing carbon–climate dynamics is reframed as a network congestion control problem, incorporating critical concepts to highlight gaps in current scientific approaches to climate feedback management. **Third**, an algorithm based on an implicit open–loop control assumption, incorporating the need for continuous adjustments when discrepancies arise between targets and actual measurements, is introduced. Additionally, taking into account nonlinear behavior and feedback from an international carbon monitoring system, the authors show how the task of regulating the global carbon cycle may be viewed as an abstracted network congestion problem using a reduced complexity model. Finally, a simulation scenario demonstrating how closed–loop control could be developed to more effectively manage the carbon–climate structure is presented.

## 1 Introduction

One of the most important global issues of the present time is climate change, which is unquestionably a result of human activity. The term “climate change" refers to a general alteration of the earth’s climate, which includes changes to land and ocean temperatures as well as precipitation and snowfall amounts. The following are some pertinent climate–related factors: rays of the sun, earth, the oceans, the wind, the rain, the snow, the woods, the deserts, the savannas, and all human activity. Before industrialization, greenhouse gases were naturally balanced and their impact on climate was relatively stable. The greenhouse effect, which prevents the earth’s atmosphere from reflecting the sun’s heated infrared radiation, has been the primary driver of climate change since industrialization. The intricate relationships between several natural elements and human–caused impacts make it difficult to study and predict climate dynamics [1]. To deal with this complexity, researchers have developed novel mathematical models that can capture the uncertainties and non-linear interactions inherent in the climate system. One such model that has gained recognition for its ability to convey intricate relationships, adjust to shifting circumstances, and provide insightful predictions in the context of climate change is the *T–S (Takagi–Sugeno) fuzzy* framework [2]. Based on fuzzy logic principles, the *T–S fuzzy model* offers a structured framework for representing complex systems by breaking them down into the smallest, locally valid sub–models [3]. A comprehensive representation of the entire system is then produced by combining these sub–models. Given the complex interactions between multiple components in the subject of climate change, the *T–S fuzzy model*’s capacity to capture nonlinearities and uncertainty proved crucial. This paradigm provides a means of representing complex processes using fuzzy sets and linguistic variables, which is ideally suited to the inherently vague and imprecise nature of climate data.

Earth’s climate dynamics are defined by a variety of elements, such as temperature, precipitation, concentrations of greenhouse gases, ocean currents, and others. These variables exhibit feedback loops, thresholds, and nonlinear interactions that are challenging to incorporate into conventional linear models [4]. By segmenting the input space into numerous parts, each of which is linked to a locally dependable linear sub-model, the *T–S fuzzy model* gets around this problem. This localized modeling approach can be used to depict nonlinear behaviors for certain ranges of input variables. In addition, due to natural fluctuations, lack of data, and changes in human activities, there are still unknown factors contributing to climate change. The *T–S fuzzy* framework [5] is highly effective at handling uncertainties, as it incorporates fuzzy set theory to explicitly model imprecision. This competency is crucial when working with climate projections, as evaluating uncertainty is necessary for making wise decisions. This study of climate change modeling using the *T–S fuzzy* framework covers the fundamental concepts of the *T–S model*, its application to climate systems, and its advantages over traditional modeling approaches. By accepting the inherent complexity and uncertainty of climate dynamics, the *T–S fuzzy model* opens up new avenues for enhancing our understanding of climate change, assisting with well–informed policy decisions, and fostering a sustainable future.

In order to combat atmospheric systems, recent research emphasizes the expanding convergence of energy systems, environmental sustainability, and artificial intelligence. In their investigation of cellular and biochemical pathways, Li et al. [6] offered insights into regulatory mechanisms that may have consequences for climate–resilient systems and bioengineering. Multi-source information fusion strategies were developed in [7] and can improve predictive environmental models by increasing accuracy in the face of ambiguity. By directly connecting advanced analytics to the monitoring and mitigation of climate–related pollutants, Wu et al. [8] highlighted the potential of geospatial AI in air pollution prediction. In order to maximize the integration of renewable energy sources and minimize emissions, Meng et al. [9] suggested low–carbon dispatching solutions for integrated energy systems. In their evaluation of district sharing plans for heating and cooling using ground source heat pump (GSHP) systems, Zhang et al. [10] highlighted workable ways to reduce carbon emissions in urban energy use. In order to show how large-scale interactions affect global climate variability, Zhu et al. [11]looked at the effects of tropical regions on Arctic climate patterns. Lastly, in [12] looked at how cloud computing may decouple urban carbon footprints and provided solutions for sustainable urbanization. When taken as a whole, these works are significant because they tackle climate change from various but complementary angles, such as urban carbon management, predictive AI, and system optimization. This lays the groundwork for creating integrated frameworks that integrate sustainability objectives, technology, and policy.

### 1.1 Significance of T-S fuzzy model for Climate Change

The use of *Takagi–Sugeno Fuzzy Models (T–S models)* in climate change study is crucial to comprehending the many facets of this worldwide problem. By addressing nonlinear linkages within climate systems, these models offer a unique perspective and transcend the limitations of traditional linear methodologies. By effectively managing uncertainty, they address the inherent ambiguity in climate data [13]. It is also essential to incorporate expert information, which allows climate scientists to offer qualitative insights and increase forecast accuracy. *T–S models* have a revolutionary effect on a number of applications, from predicting short–term climate changes to assessing the potential effects on sectors like agriculture, water resources, and energy. Their ability to facilitate scenario analysis and policy evaluation empowers decision-makers to make educated choices, directing society toward effective mitigation and adaptation strategies for climate change [14]. In a world grappling with the severity of climate change, *Takagi–Sugeno Fuzzy Models* are an essential tool that offer insights into the complex interplay of variables and uncertainties affecting our planet’s future [15].

**Fig 1:**
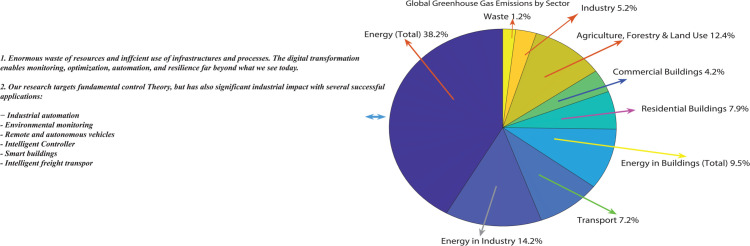
Emissions of greenhouse gases worldwide by sector [16].

A comprehensive overview of *global greenhouse gas (GHG)* emissions by sector for *2016*–when total emissions reached 49.4 billion tonnes of *CO*_2_–is shown in Fig 1’s block diagram. With 38.2% of global emissions, the energy sector is the largest contributor, as the graphic makes clear. Energy consumption in buildings (14.2%), and transportation (7.2%), and are the main sources of energy in this sector, with residential and commercial buildings accounting for the majority of contributions. Livestock, deforestation, and soil management are the main drivers of the 12.4% contribution from the Agriculture, Forestry & Land Use sector. Waste (1.2%) and Industry (5.2%), on the other hand, are lesser but nonetheless important sources. The graphic also shows possible ways to improve resource efficiency through digital transformation, which makes it possible for improved automation, optimization, and monitoring. *GHG* emissions worldwide by sector (derived from [Ref [16]). The baseline data supporting the fuzzy control method used in this investigation is shown in this picture. The urgent need for adaptive control strategies–which are further explored in *Sects* 3–6, which is demonstrated by the significant contributions from the energy, building, and transportation industries. In order to improve sustainability and lower emissions, the green–highlighted section describes significant research applications such as smart buildings, intelligent warehouses, and wireless industrial automation. Industries may fix inefficiencies, reduce their environmental effect, and aid in the fight against climate change by incorporating these cutting–edge solutions. For more comprehensive review, readers can refer the references [17,18].

**Fig 2:**
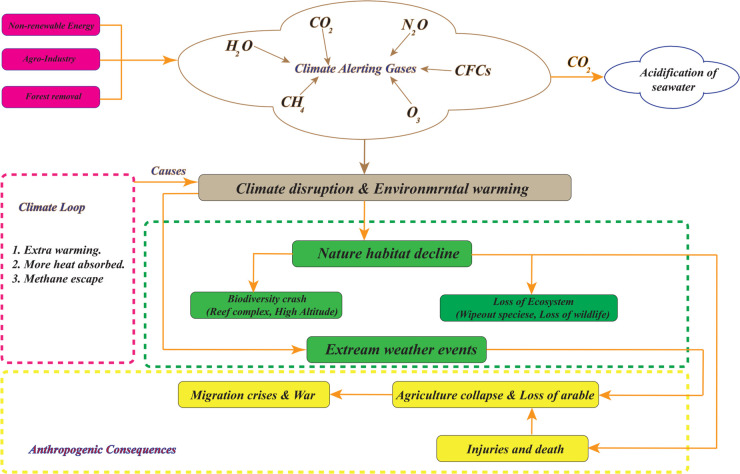
Causes and effect of Climate change and Global warming.

Climate mechanism and global warming are complicated environmental issues brought on by both natural and human–caused causes. The interrelated causes, feedback systems, and ensuing effects on the environment and human society are depicted in this block diagram. The main contributors to climate change include deforestation, agriculture, and the use of fossil fuels, all of which increase the emission of greenhouse gases including nitrous oxide, carbon dioxide, and methane. These gases exacerbate the greenhouse effect and fuel global warming by trapping heat in the Earth’s atmosphere. The Fig 2 emphasizes the function of feedback loops, wherein thawing permafrost, increasing water vapor, and decreased snow cover all contribute to the acceleration of climate change. Environmental effects of these changes include habitat loss, ecosystem collapse, biodiversity loss, increased weather, glacial retreat, and sea level rise. Human problems including agricultural failure, flooded towns, population displacement, and health hazards are further exacerbated by these environmental repercussions. The root causes and effects of global warming and climate change. The nonlinear feedback loops (such as water vapor amplification and permafrost thawing) that are subsequently modeled using fuzzy rules (Table 1) and assessed in simulation tests (Figs 12–20) are highlighted in this schematic. It is clear from comprehending the chain reactions shown in this graphic that climate change is a complex problem that needs immediate attention to lessen its profound effects on ecosystems and human culture.

**Table 1:** Fuzzy rules for global climate system.

Rule No.	IF Condition 1	IF Condition 2	THEN Result
1	Temperature is High	*CO*_2_ is High	Chance of High Temperature
2	Temperature is Low	*CO*_2_ is Low	Chance of High Snowfall
3	Temperature is High	Terrestrial biosphere ($\xi_{T}$) is High	Increase the risk of Species Extinction
4	Temperature is Low	Terrestrial biosphere ($\xi_{T}$) is Low	Decrease the risk of Species Extinction
5	Sea surface is High	Terrestrial biosphere ($\xi_{T}$) is Low	Increase in Biodiversity
6	Sea surface is Low	Terrestrial biosphere ($\xi_{T}$) is High	Decrease in Biodiversity
7	Wind speed is High	Surface ocean ($\xi_{S}$) is Large	Structural damage in Buildings & Trees
8	Wind speed is Low	Surface ocean ($\xi_{S}$) is Low	No effect
9	Wind speed is High	Climate risk High	Increase Energy sources
10	Wind speed is Low	Climate risk Low	Decrease Power output

Applying *T–S fuzzy models* in climate change has practical significance. These models offer an easy and efficient approach to incorporate uncertainty in input and output variables, such as projected *greenhouse gas (GHG)* emissions and temperature increases. T–S fuzzy models help capture the relationships between *GHG* concentrations and temperature variations by using linguistic rules derived from emission scenarios and climate models [19,20]. Fuzzy models can also account for climate sensitivity uncertainty, giving a more thorough evaluation of temperature rises [21]. *Decision–makers* can test alternative strategies and analyze their results at different levels by using fuzzy models to measure regional sustainability development under climate change [22]. In general, *T–S fuzzy models* provide useful tools for making decisions and comprehending the effects of climate change.

**Fig 3:**
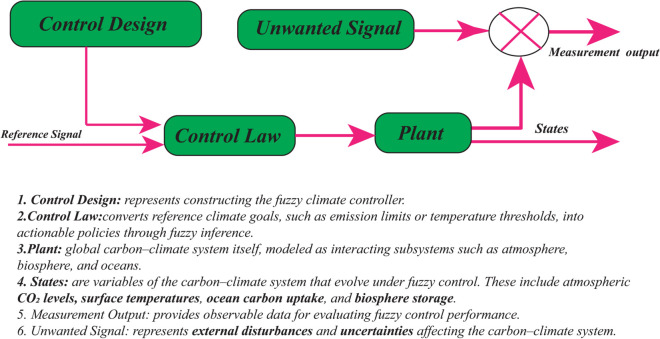
Traditional open–loop controller representation for controlling dynamical systems and their counterpart strategies in managing carbon climate systems.

**Fig 4:**
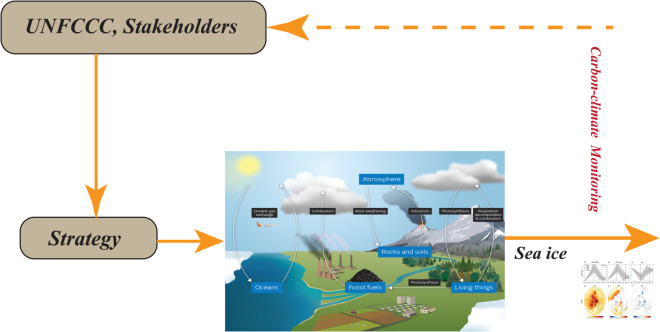
Conventional description of a closed-loop controller for controlling dynamical systems and their counterpart strategies in managing carbon climate systems.

**Fig 5:**
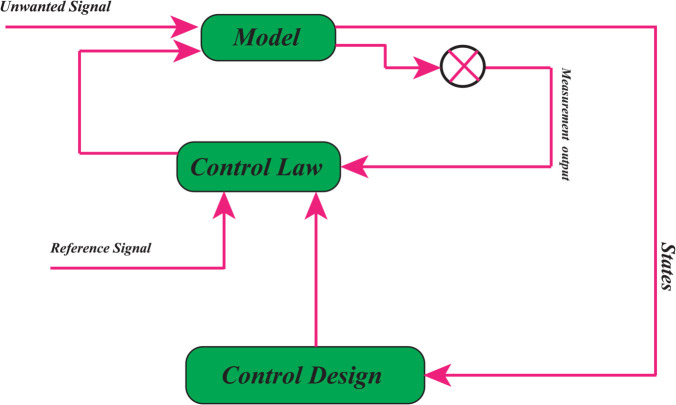
Policies for open-loop control and climate change mitigation for controlling dynamical systems and their counterpart strategies in managing carbon climate systems.

**Fig 6:**
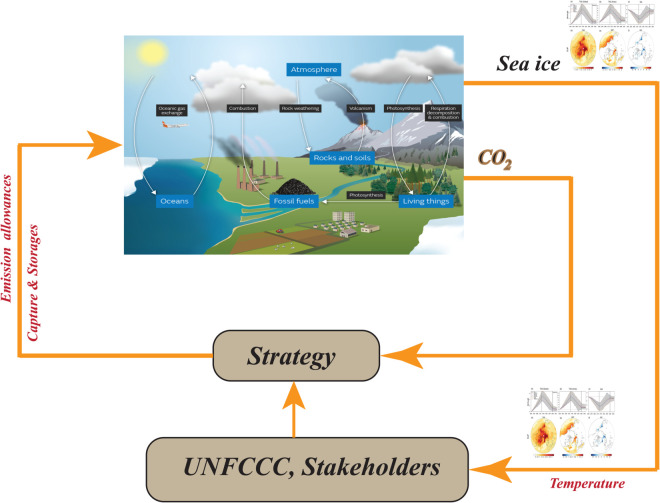
Closed–loop control, measurements design and assessment of policies pertaining to climate change adaptation.

In [23], two strategies for controlling complex dynamical systems are outlined: open–loop control, also known as pre-computed control (OCLC), and closed-loop control. As shown in Figs 3 and 4, feedback (closed–loop) control adjusts actions based on observations of the system state, allowing it to correct deviations and account for random perturbations caused by noise or model uncertainties [24]. In cutting–edge research on carbon–climate policy, however, open–loop models are often employed (See Figs 5 and 6). Studies on carbon–climate feedback heavily rely on scenario–driven analyses using long–term emission pathways to forecast climate impacts [25], and their results significantly shape climate mitigation strategies aligned with particular scenario trajectories [26].

*Control theory* plays an essential role in climate change mitigation, providing a framework for analyzing and managing the complex systems that influence Earth’s climate. Encompassing a range of methodologies and techniques, it enables the regulation of key drivers such as greenhouse gas emissions, deforestation, and industrial pollution. By applying control principles, policymakers and scientists can design strategies and interventions that guide environmental systems toward sustainability and resilience. This multidisciplinary approach draws on engineering, mathematics, and environmental sciences to develop effective mechanisms for mitigating climate change at a global scale. The primary contributions can be outlined as follows:

- Introduces a *novel fuzzy logic–based mathematical* representation of the carbon–climate system to formalize the closed–loop control problem.
- Develops a segmented mathematical representation of the global carbon system that highlights characteristics such as dynamical stability, demonstrating through simulations that fuzzy logic rules can effectively regulate fossil fuel demand and carbon transfer to geological reserves like the deep sea.
- Derives a mathematical decision-making guideline from system demand and internal dynamics, enabling adaptive carbon cycle management as an alternative to long–term scenario–based recommendations, with policy decisions supported on shorter time scales.

The subsequent part of this article is organized as follows. Sect 2 delves into the principles of Control Theory in the context of *Climate Change Mitigation*. Our mathematical depiction of the *Global Carbon Climate Model (GCCM)* is outlined in Sect 3. Sect 4 elaborates on a novel fuzzy model for the *Global Climate Model (GCM)*. The interconnected control methodology for the *Global Climate System (GCS)* is expounded in Sect 5. Lastly, the ultimate findings are disclosed in Sect 6.

## 2 Concepts of control theory in the Mitigation of Climate Model

*Control theory* encompasses many concepts, strategies, and techniques involving optimization ideas. Although optimization concepts have long been applied in areas such as economics (e.g., inflation control) and in recognizing the benefits of mitigating global warming, applications of contemporary control theory to climate systems remain relatively new. In addition, the concepts of optimization were instrumental in the creation of *Integrated Assessment Models (IAMs)* [27], which can provide optimal prescriptions that can be used as policy responses. Their central tenet is cost–benefit optimization, which incorporates societal costs of climate impacts to maximize social welfare by exploring a space of future emission trajectories with associated environmental effects [28]. Emissions time trajectories offer data for policymakers, but they lack official feedback and corrective procedures grounded in actual observations.

Few studies have employed coupled carbon–climate AI models, which is surprising given their significant potential for modeling and managing the global carbon cycle. In contrast to Solar Radiation Management (SRM), which faces significant uncertainties regarding environmental impacts and may never be fully deployed, substantial progress has been made in reducing emissions and sequestering carbon in long–term reservoirs. To the best of current knowledge, formal closed–loop control concepts have not been applied within emission trading policy frameworks based on fuzzy model. Furthermore, very few scientific studies have examined feedback regulation processes in the global carbon cycle using similar ideas.

Using locally linear sub–models, the data–driven T–S fuzzy identification described above provides a systematic approach to approximating nonlinear systems. We apply this method to the global carbon–climate system in the next section. Specifically, climatic drivers such as greenhouse gas emissions, solar radiation, and biosphere feedbacks serve as fuzzy inputs, while the model’s state variables–carbon reservoirs and surface temperature–are represented as outputs of fuzzy sub–models. The nonlinear dynamics outlined in Eq (1) can thus be expressed within the T–S fuzzy framework.

## 3 Mathematical representation of the Global Climate Model

At any particular point in time domain (t,ℛ), the *global carbon–climate system* can be represented as a vector $s(t)\in {ℛ}_{n}$ with components representing *carbon levels* and *temperatures* across earth system reservoirs. The function *s*(*t*) representing the state at a given time span can be formally articulated as the outcome of a specific organizing $ϝ:(s_{0},\nu )\mapsto s$ with initial condition $s_{0}:=s(t_{0})$ and ν(t) denotes the *driving signal* in term of vector. For instance, one may consider ϝ as a representation of the Earth system, incorporating the initial condition *s*_0_ along with a set of time–varying driving parameters ν(t) defined over the time span $𝐓=[t_{0},t_{max}]$ and produces the complete evolution of the state *s*(*t*) over the time interval **T** within the overarching *carbon–climate model*. Although ϝ can be expressed in various ways, the authors will confine its definition in this case study to an *initial value problem (IVP)* for an interconnected set of ordinary differential equations that represent carbon accumulation within *Earth reservoirs* denoted as ${\mathbb{C}}_{E}(t)$, and *surface temperatures* expressed through block matrices as ${𝕋}_{S}(t)$.

$$\dot{s}(t)=\left[\begin{array}{c} {\dot{\mathbb{C}}}_{E}(t)\\ {\dot{𝕋}}_{S}(t) \end{array}\right]=\left[\begin{array}{c} 𝔹({\mathbb{C}}_{E},{𝕋}_{E},t).{\mathbb{C}}_{E}+s(t)\\ f({\mathbb{C}}_{E},\mathbb{R},t) \end{array}\right]$$ (1)

In the next section, the authors will present the new concept of *T–S fuzzy* model for the *climate control plant*.

**Remark 1.** *The climate model presented here is the particular application area in which the generic T–S fuzzy methodology of* Sect 2 *is used; it is not separate from the fuzzy identification framework. By approximating the temperature feedback functions f(·) and nonlinear transfer rates (matrix 𝔹) in* Eq (1)*, the fuzzy sub–models integrate data–driven fuzzy identification into the climate control environment.*

## 4 New fuzzy model for Global Climate Model

We model the global carbon–climate plant as a nonlinear augmented system (1), building on the fuzzy identification concepts discussed in Sect 2. Fuzzy rules convert qualitative climate knowledge (such as "high *CO*_2_ with high temperature → accelerated warming") into quantitative model structure, and T–S fuzzy sub–models derived from available data capture the compartmental dynamics (carbon exchange among atmosphere, biosphere, and ocean). Our focus here is still on carbon–climate dynamics, but the framework is broad enough to be applied to other environmental problems. The above model (1) illustrates how *Fuzzy Models* can be used to predict changes in climate environment due to rising sea levels. The main steps have been listed below:

**Scenario**: Fuzzy carbon climate prediction under sea level rise in the following way:

**Step 1 (Variable Identification)**: *Sea level rise (SLR)* and *wave energy (WE)* are two climatic factors to consider. Define the terms "*low*," "*medium*," and "*high*," as well as their membership criteria, for each.

**Step 2 (Fuzzy Rules)**: Specify the linguistic effects of *SLR* and *WE* on fuzzy carbon climate by developing fuzzy rules. Consider this:

- If both *SLR* and *WE* are high, then there will be severe fuzzy carbon climate.
- If both *SLR* and *WE* are low, then there will be little fuzzy carbon climate.

**Step 3 (Fuzzy Inference System)**: In order to forecast the severity of fuzzy carbon climate based on the current sea level rise and wave energy conditions, apply the fuzzy rules. The model predicts severe fuzzy carbon climate (0.65), for instance, if *SLR* is *medium (0.6)* and *WE* is *high (0.7)*.

**Step 4 (Defuzzification)**: From the fuzzy output, generate a precise value that, under the given circumstances, indicates the extent of fuzzy carbon climate. This can be achieved by taking a weighted average of the fuzzy rule’s output values.

### 4.1 From compartmental dynamics to a T–S fuzzy model

Let $\xi (t)={\left(\begin{array}{c} \xi_{A}(t)\\ \xi_{T}(t)\\ \xi_{S}(t) \end{array}\right)}^{T}$, denote the carbon stock in atmosphere, terrestrial biosphere, and surface ocean (*PgC*). The Eqs (12)–(9) give:

$$\dot{\xi }(t)=𝔹(\xi ,t)\;\xi (t)+m(t),\;m(t)=[\lambda_{F\to A}(t),\;0,\;\lambda_{D\to S}{]}^{⊤},$$ (2)

with entries of 𝔹(ξ,t) defined by the fluxes $\lambda_{X\to Y}$ (Eq (12)) with parameters $\gamma_{1},\gamma_{2}$. We define the scheduling vector:

$$z(t)=[z_{1}(t),z_{2}(t),z_{3}(t)]=\left[\frac{\xi_{A(t)}}{700},\;\frac{\xi_{T(t)}}{3000},\;\frac{\xi_{S(t)}}{1000}\right],$$ (3)

where augment *z*(*t*) with exogenous radiative forcing.

#### 4.1.1 Partition and membership functions.

**Fig 7:**
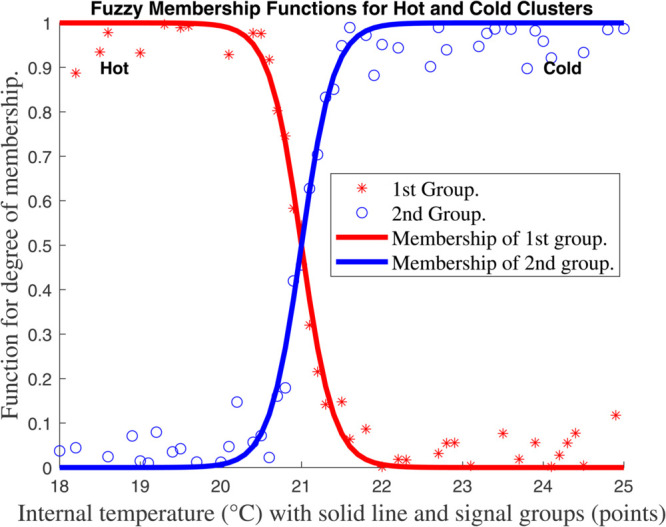
Membership function for global climate model for internal temperature (^∘^*C*) with solid line and signal groups (points).

**Fig 8:**
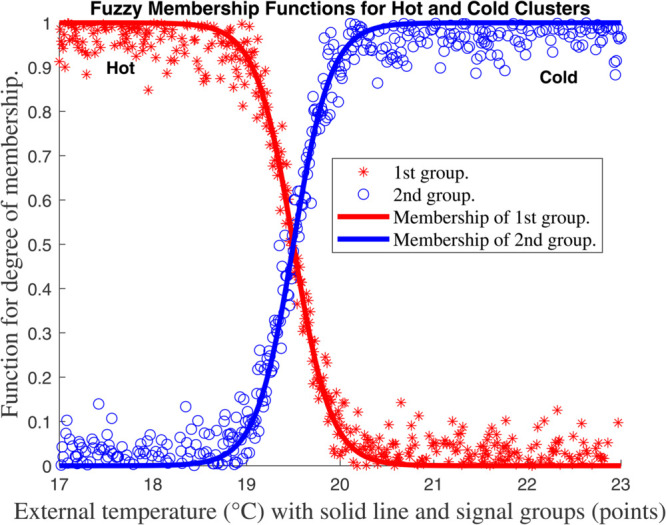
Membership function for global climate model for external temperature (^∘^*C*) with solid line and signal groups (points).

**Fig 9:**
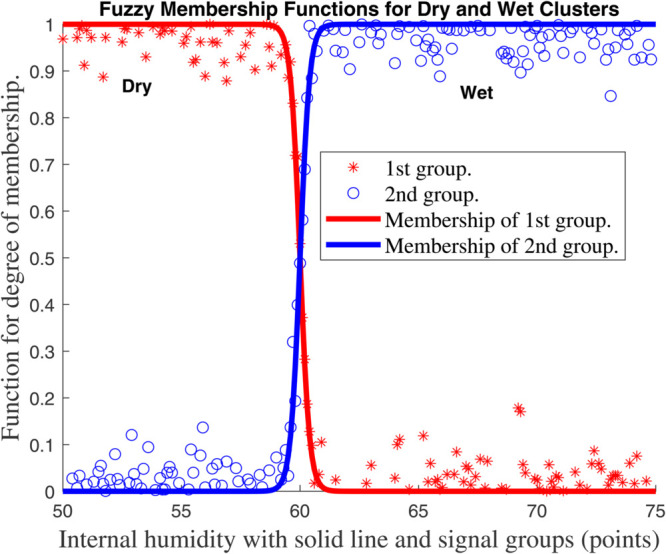
Membership function for global climate model for Internal humidity with solid line and signal groups (points).

**Fig 10:**
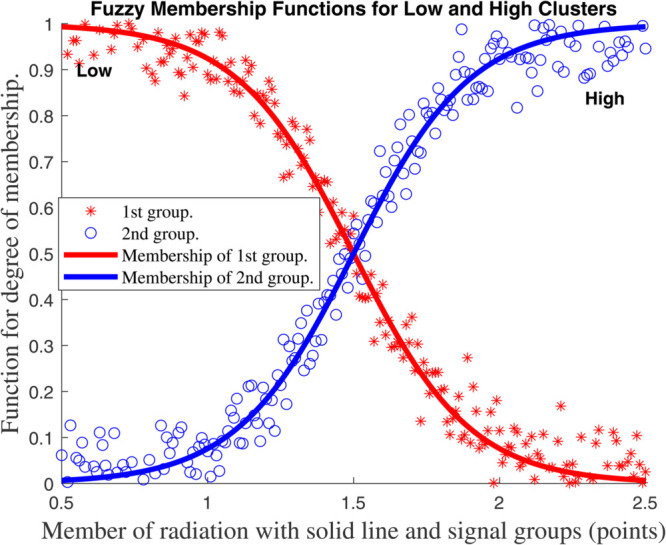
Membership function for global climate model for member of radiation with solid line and signal groups (points).

For each *z*_*k*_ we select *n*_*k*_ overlapping fuzzy sets (e.g., *Low/High* triangles as in Figs 7, 8, 9, and 10, with membership $\mu_{k,j}(z_{k})$. The *i*–th rule corresponds to one set per *k*, with normalized weight

$$h_{i}(z)=\frac{\prod_{k=1}^{3}\mu_{k,\sigma_{i}(k)}(z_{k})}{\sum_{\ell =1}^{r}\prod_{k=1}^{3}\mu_{k,\sigma_{\ell }(k)}(z_{k})},\;r=\prod_{k}n_{k},$$ (4)

where $h_{i}(z)\geq 0$ and $\sum_{i=1}^{r}h_{i}(z)=1$.

#### 4.1.2 Local affine sub-models.

Within each fuzzy region, the state–dependent coefficients in 𝔹(ξ,t) are approximated at the region’s nominal point, yielding:

$$\dot{\xi }(t)={𝔸}_{i}\;\xi (t)+{𝔹}_{i}\;u(t)+m(t),$$ (5)

with


$${𝔸}_{i}:=𝔹({\bar{\xi }}^{\left(i\right)},{\bar{t}}^{\left(i\right)})$$

Here u(t)=ψ(κ(t)) is the control input, and *p*(*t*) is the disturbance or demand term. The global T–S fuzzy model is then obtained as:

$$\dot{\xi }(t)=\sum_{i=1}^{r}h_{i}(z(t))\left({𝔸}_{i}\;\xi (t)+{𝔹}_{i}\;u(t)+m(t)\right).$$ (6)

With $z_{1}=\xi_{A}/700$ (*Low/High*); $z_{2}=CO_{2}$ (*Low/High*); $z_{3}=Terrestrial\;biosphere\;\xi_{T}$ (*Low/High*); $z_{4}=Sea\;surface\;\xi_{S}/1000$ (*Low/High*); $z_{5}=Wind\;speed$ (*Low/High*); and $z_{6}=Climate\;risk$ (*Low/High*), ten rules are obtained:

- ${ℜ}_{1}$: If *z*_1_ is **Low** and *z*_2_ is **Low**, then **chance of high temperature.**
- ${ℜ}_{2}$: If *z*_1_ is **Low** and *z*_2_ is **Low**, then **chance of high snowfall.**
- ${ℜ}_{3}$: If *z*_1_ is **High** and *z*_3_ is **High**, then **increase of risk of species extinction temperature.**
- ${ℜ}_{4}$: If *z*_1_ is **Low** and *z*_3_ is **Low**, then **decrease of risk of species extinction temperature.**
- ${ℜ}_{5}$: If *z*_4_ is **High** and *z*_3_ is **Low**, then **increase in biodiversity.**
- ${ℜ}_{6}$: If *z*_4_ is **Low** and *z*_3_ is **High**, then **decrease in biodiversity.**
- ${ℜ}_{7}$: If *z*_5_ is **High** and *z*_4_ is **High**, then **structural damage in buildings & trees.**
- ${ℜ}_{8}$: If *z*_5_ is **Low** and *z*_4_ is **Low**, then **no effect.**
- ${ℜ}_{9}$: If *z*_5_ is **High** and *z*_6_ is **High**, then **increase energy sources.**
- ${ℜ}_{10}$: If *z*_5_ is **Low** and *z*_6_ is **Low**, then **decrease in power output.**

This framework ties the compartmental dynamics (Eqs (5)–(7)), membership functions (Fig 7, 8, 9, and 10, and the control law ψ(κ) into a unified T–S fuzzy representation of the carbon–climate system.

**Remark 2.** *The main contribution of this research has been the following: • For compartmentalized reservoirs (atmosphere, terrestrial biosphere, and surface ocean), T–S fuzzy logic is used. • Integrating uncertainty and nonlinear dynamics into carbon feedback circuits. • Fuzzy rule development for adaptive carbon cycle management (as demonstrated in* Figs 8*–*10 *and* Table 1*.)*

Figs 7, 8, 9, and 10 show the outcomes for each input, indicating the groupings created by the fuzzy approach and the *functions for membership* of the *fuzzy plant*. To reduce the amount of rules in the *T–S fuzzy structure*, we choose a limited number of groups for every input. Four subplots depicting fuzzy membership functions for environmental variables such as temperature, humidity, and radiation are included in Figs 7, 8, 9, and 10. The link between environmental parameters and their respective membership functions is depicted in each subplot. Every subplot adheres to a similar structure, such as using red stars (^*^) to symbolize data points. In other words, those indicate data points from the first group (e.g., ’Hot’, ’Dry’, or ’Low’). ’Cold’, ’Wet’, or ’High’ data points are represented by blue circles (*o*) in category two. Additionally, the membership functions for the first and second groups are shown by solid red and blue lines, respectively.

Every graphic exhibits the fuzzy logic properties of soft transitions between two states (hot/cold, dry/wet, etc.). This is a basic feature of fuzzy logic that deals with overlapping conditions and uncertainty. Since it can be challenging to set exact thresholds, these fuzzy membership functions are commonly used in *heating, ventilation, and air conditioning (HVAC) systems*, *climate management*, and *smart building automation*. The system’s adaptable capability, which is essential for practical applications, is demonstrated by the small fluctuation in crossover points between internal and external temperatures. Although there is a lot of noise in the red and blue data points, the system’s capacity to filter noise while still maintaining efficient environmental control is shown by the smooth membership functions. A clearly specified fuzzy logic system for climate and environmental control is depicted in this collection of charts. The data sets’ logical grouping, adaptive crossover points, and seamless transitions demonstrate a reliable technique for simulating overlapping or uncertain situations that are frequently present in real-world settings.

To establish the fuzzy rules for the climate plant, we have some certain range for climate change model. For the variable identification, we assume with a range of 0 → 1, let *SLR* and *WE* stand for sea level rise and wave energy, respectively. Fuzzy rules can be modeled as:

- *SLR* must be high (0.8) and *WE* must also be high (0.7) for fuzzy carbon climate to be severe (0.75).
- In the event where both *SLR* and *WE* are low (0.2 and 0.3, respectively), fuzzy carbon climate is minimal (0.25).

Under the existing circumstances, the fuzzy inference system combines these criteria to estimate the severity of fuzzy carbon climate. This example shows how *T–S models* can forecast how climatic factors would affect it. These models, which make use of fuzzy logic to represent the intricate interactions between sea level rise, wave energy, and erosion susceptibility, provide light on the possible threats that climate change may pose to coastal communities. From Eq (1), the *global carbon cycle* can be seen to operate like a nonlinear augmented system whose outputs depend on time [29,30] which in turn influences its temperature nonlinearly. This system represents the active part of the carbon cycle. The matrix 𝔹 presents all transfer rates between system compartments and all mass inputs into it; its vector s accounts for mass inputs to it. A portion of its activity as a component of the carbon cycle is represented by this compartmental mechanism. Therefore, in terms of ocean sediments and geological reservoirs, the atmosphere, terrestrial biosphere, and ocean are open in [31]. The temperature of the surface functions *f* are dependent on the state of carbon cycle activities, solar radiation, and other radiative forcing agents ℝ [32]. Some presumptions are listed below before moving on to the core *controllability* and *stability*. In Sect 5, the authors expand the method to a worldwide carbon–climate control network, using the fuzzy model presented here as the basis for building interconnected control strategies.

**Assumption 1.** *All diagonal elements are negative.*

**Assumption 2.** *All other variables other than diagonal elements entries are positive.*

**Assumption 3.** *Sum of all columns are positive.*

## 5 Novel approach for control theory interconnected Global Climate–Carbon Network

In this section, the authors apply current control frameworks to the global climate–carbon system (e.g., [36] on congestion control in compartmental networks). Our contribution is to adapt these concepts to the nonlinear, stochastic, and multi–scale dynamics of the Earth’s carbon cycle, whereas previous work has mostly concentrated on artificial or isolated systems. In particular, the authors combine a compartmental carbon–climate model with fuzzy logic–based membership rules, allowing for adaptive control of carbon flows and greenhouse gas emissions in the face of uncertainty. By creating a closed–loop, feedback–oriented strategy that recommends shorter-horizon, adaptive control actions, this fusion enables us to go beyond long–term, scenario–driven projections. Therefore, we have developed completely new mathematical control laws, but rather because we have extended and reframed existing approaches for use in climate feedback management, where socioeconomic decision horizons, nonlinearities, and incomplete observability present particular difficulties. In order to address the issue of controlling the *global climate–carbon network*, we will now enhance the model of our plant to incorporate control signals denoted as *u*(*t*) and noise represented as *p*(*t*). The original mapping function ${ϝ}_{c}$ will now involve the consideration of two additional variables.

$${ϝ}_{c}:=(s_{0},\nu ,u,p)\mapsto s$$ (7)

The expression ${ϝ}_{c}$ may be extremely complex, encapsulating the climate mechanism’s non-autonomous, unpredictable, multi–scale, and stochastic properties. We now isolate in *u*(*t*) parameters that are controllable, such as emissions from fossil fuels and decreases in emissions, carbon storage in biological products and earth’s reservoirs, and radiation from the sun management, even though ν(t) still represents outside factors that affect the system’s behavior, such as variations in solar activity or Earth’s rotation. Additionally, the authors distinguish between model unreliability and process disturbance using the vector–valued measure *p*(*t*).

We usually only have access to a limited collection of data and measurements *y*(*t*) to comprehend the framework, and we frequently do not know the full state *s*(*t*) of the entire system. The findings, which come from a measurement network, provide information on a number of factors, including gross manufacturing, atmospheric *CO*_2_ concentrations, and atmospheric surface temperature. We now provide a mapping between these observations and the state of the system.

$${ϝ}_{y}:=(s,u,\omega )\mapsto s$$ (8)

From the above Eq 8), the outcome may be influenced by the inputs *u*(*t*), which in turn can be influenced by measurement noise ω(t). The combination of ${ϝ}_{c}$ and ${ϝ}_{y}$ forms our model, which is impacted by the inputs *u*(*t*) and output (*y*(*t*)) has been observed. Consequently, the task that society is confronted with is to ascertain the necessary course of action for *u*(*t*) in the coming years, such as for t∈[2020,2100], to mitigate the risk of hazardous warming.

**Remark 3.** *To effectively address climate change, certain key variables and parameters must be controlled. These can be grouped into two main categories:* **Emissions of greenhouse gases** *and* **Climate Mitigation Techniques***. Here’s a detailed breakdown:*

- *Greenhouse Gas Emissions (GHGs).*
- *Climate Mitigation Techniques.*

*The primary causes of the burning of fossil fuels (power plants, automobiles), industrial operations, deforestation, and changes in land use have an impact on control methods through the use of renewable energy, enhanced energy efficiency, forest restoration, and tree planting. On the other side, carbon sequestration can be described by planting trees and restoring forests to absorb *CO**_*2*_ *. The effectiveness is rely on the highly in suitable regions, with long-term benefits. Secondly, Soil Carbon Sequestration have capability to enhancing soil management practices to increase carbon storage, which significant effect on potential, especially in agricultural regions. This factor can further improving the efficiency of energy use in buildings, transportation, and industry. Reduces energy demand and emissions, cost-effective with immediate benefits. Effective control of these variables and parameters involves a combination of reducing GHG emissions, enhancing carbon sequestration, and exploring innovative climate mitigation techniques. Each strategy’s effectiveness can vary based on geographical, technological, and economic factors. A comprehensive and integrated approach, supported by strong policies and international cooperation, is essential to achieve significant climate mitigation outcomes.*

### 5.1 Designing control systems involves the process of creating control laws

Measurements are mapped to control actions through a control law 𝕂. Control laws are typically designed by tuning parameters or design structures using properties or system behavior. The majority of previous studies of closed-loop control have focused on controlling climate or socio-ecological systems [33,34]. In all of these studies, linear or linearized models are employed and/or linear feedback control methods are utilized, with *proportional–integral controllers* and their modifications being the most commonly used. Many applications have been successfully implemented with *PI controllers*. The significant limitation of this method is the requirement that the system remain near the point at which it has been linearized, i.e., the point where the nonlinear system was linearized. Climate change, however, is by its very nature nonlinear. It is possible to exploit nonlinear interactions for control by linearizing around a specific operating point, eliminating critical nonlinear interactions, which are critical for prediction. It is also important to note that disturbances and uncertainties are likely to cause significant changes to the system and render the controller invalid as a result. A *model–based control approach* appears to be promising and can have a greater impact given the existence of realistic climate models. Using a simple model as part of the *model–based control strategy*, [35] provide a good example of this concept. In addition to minimization or maximization of the control objective, The control work as established by the control rule 𝕂 itself may likewise be penalized by *u*(*t*). When using a compartmentalized nonlinear closed–loop controller, [36] showed how to prevent mass congestion in compartmental networks automatically. To maximize the network’s throughput, reservoirs that temporarily store carbon can be designed and implemented to maximize the carbon management strategy. The controller can figure out how much mass can enter the network and calculate mass allowances. We think this control mechanism can be helpful as a model worldwide system for reducing climate change and the management of carbon.

**Algorithm 1 FCC–I: Fuzzy closed-loop carbon–climate control.**

**Given inputs** $T=\{t_{0},\ldots ,t_{\max}\}$, Δt, initial state $\xi (t_{0})=[\xi_{A}^{0},\xi_{T}^{0},\xi_{S}^{0}{]}^{⊤}$, demand p(t)≥0, rule base ℜ, membership functions (MFs), flux maps $\lambda_{•\to •}(\cdot )$, and constants $\lambda_{S\to D},\lambda_{D\to S}$.

**Choose** bounds for tunable $\Theta =\{\gamma_{1},\gamma_{2},\kappa_{\max},MFthresholds/widths\}$.

1: **Phase 1: Control variable configuration**

2: **ForAll** θ∈Θ **do** ⊳ *θ* groups $\gamma_{1},\gamma_{2},\kappa_{\max}$, MF cut–points

3:   **For** $k=1,2,\ldots ,T_{\max}$ **do**

4:   Randomly draw MF cut–points and $\kappa_{\max}$ from *θ*-bounds

5:   Using current $\left(\gamma_{1},\gamma_{2}\right)$, compute one-step flux maps: $\lambda_{A\to T}\;=\;60{\left(\xi_{A}/700\right)}^{\gamma_{1}}$, $\lambda_{A\to S}=100(\xi_{A}/700)$, $\lambda_{T\to A}=60(\xi_{T}/3000)$, $\lambda_{S\to A}=100{\left(\xi_{S}/1000\right)}^{\gamma_{2}}$

6:   Build fuzzy antecedents from MFs; fire rules *R* to get weights; defuzzify $\to z_{fuzzy}$

7:   Define controller shape ψ(κ):


$$\psi (\kappa )=\left\{ \begin{array}{cc} g(\kappa ), & \kappa \leq \kappa_{\max}\\ 1, & \kappa >\kappa_{\max} \end{array}\right.$$

where *g* is cubic Hermite with g(0)=0, $g(\kappa_{\max})=1$, $g^{\prime }(0)=1/\kappa_{\max}$, $g^{\prime }(\kappa_{\max})=0$

8:   Compute feedback $y(t)=\lambda_{S\to D}-\lambda_{D\to S}$ and buffer update. κ←κ+Δt(y−ψ(κ)p)

9:   **Go to** previous Step.

10: **Phase 2 : Output (Control) Determination**

11: Set ψ(·) and MFs from *H*_*L*_; simulate over $\left[t_{0},t_{\max}\right]$:

12: **for do**$t=t_{0},\;t_{0}+\Delta t,\;\ldots ,\;t_{\max}$

13:   u(t)←ψ(κ(t));  $\lambda_{F\to A}(t)\leftarrow u(t)\;p(t)$

14:   ${\dot{\xi }}_{A}\leftarrow \lambda_{T\to A}+\lambda_{S\to A}-\lambda_{A\to T}-\lambda_{A\to S}+\lambda_{F\to A}$

15:   ${\dot{\xi }}_{T}\leftarrow \lambda_{A\to T}-\lambda_{T\to A}$;  ${\dot{\xi }}_{S}\leftarrow \lambda_{A\to S}-\lambda_{S\to A}-\lambda_{S\to D}+\lambda_{D\to S}$

16:   $\xi \leftarrow \xi +\Delta t\;[{\dot{\xi }}_{A},{\dot{\xi }}_{T},{\dot{\xi }}_{S}{]}^{⊤}$

17:   $y(t)\leftarrow \lambda_{S\to D}-\lambda_{D\to S}$;  κ←κ+Δt(y−up)

18: **end for**

19: **Return** tuned parameters, trajectories {ξ(t)}, control $u^{⋆}(t)$, and buffer κ(t)

## 6 Simulation test scenarios

**Fig 11:**
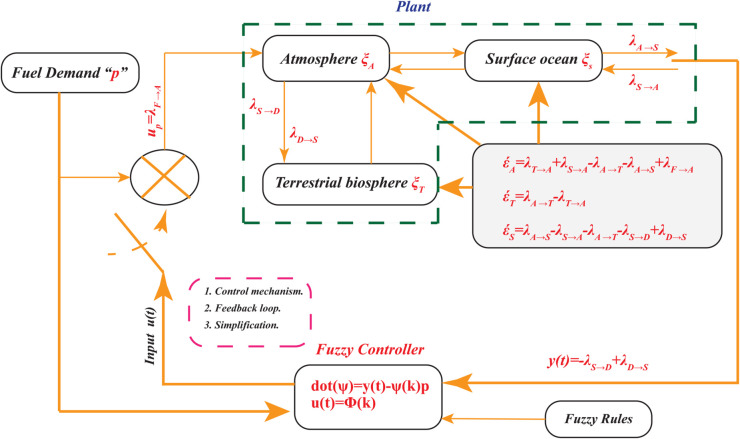
Nonlinear global carbon–cycle fuzzy control scheme using output feedback.

By contrasting the framework proposed by [36], which attempts to prevent congestion in compartmental networks, the authors demonstrate the effectiveness of *closed–loop control* in this example. The idea behind global *CO*_2_ cycling is that has been transferred across several compartments before being released into the deep ocean. Our goal is to prevent the atmospheric compartment from overflowing when we release carbon from fossil fuels. The surface of the ocean and grassland biosphere exchange carbon with the atmosphere, but they are only able to capture and digest a certain quantity of it. Fig 11 shows the behavior of net fluxes between the deep seafloor and the surface, allowing us to develop a controller that will indicate the amount of carbon from fossil fuels that may enter the atmosphere. In this case, temperature feedback is not taken into account for convenience.

This paper presents a modified version of [37,38] simple nonlinear global carbon cycle model. This can be seen in Fig 11 as the dashed rectangle within the green box, which represents the following three carbon components:

1. Atmospheric ($\xi_{A}$).
2. Terrestrial biosphere ($\xi_{T}$).
3. Surface ocean ($\xi_{S}$).

Three carbon compartments are contained in the model: atmosphere $\left(\xi_{A}\right)$, terrestrial biosphere $\left(\xi_{T}\right)$, and surface ocean $\left(\xi_{S}\right)$. Deep ocean and fossil fuel reserves are designated by the letters *D* and *F*. The amount of Carbon contents is always expressed in *PgC* and fluxes in *PgC* year^−1^. Adding carbon to the system is the result of two external fluxes. As $\lambda_{D\to S}$ is constant, As a result of the combustion of fossil fuels, *CO*_2_ is released into the atmosphere, whereas $\lambda_{F\to A}=\lambda_{F\to A}(t)$ is depend upon the time, fossil fuel burns and releases carbon dioxide into the atmosphere. Fig 6 shows how $\lambda_{F\to A}=u(t)p$ is defined by the input control *u*(*t*) and the output human demand *p*. Surface seawater and deep ocean water are the only two places where carbon can leave the system. A flux of this kind is denoted by the number $\lambda_{S\to D}$. As a result of the model, the following fluxes exist from compartment *X* to compartment *Y*, all given in *PgC* year ^−1^.

$$\begin{array}{cc} \lambda_{A\to T}=60{\left(\xi_{A}/700\right)}^{\gamma_{1}}, & \lambda_{A\to S}=100\xi_{A}/700\\ \lambda_{T\to A}=60\xi_{T}/3000, & \lambda_{S\to A}=100{\left(\xi_{S}/1000\right)}^{\gamma_{2}}\\ \lambda_{S\to D}=45\xi_{S}/1000, & \lambda_{D\to S}=45. \end{array}$$ (9)

It is controlled by two parameters, $\gamma_{1}=0.2$ and $\gamma_{2}=10.0$ which control the fluxes between the atmosphere and biosphere, as well as between the surface ocean and atmosphere. Three ordinary differential equations can now be used to describe the model, for *t*>*t*_0_ = 1765.

$$\left\{ \begin{array}{ccc} {\dot{\xi }}_{A} & = & \lambda_{T\to A}+\lambda_{S\to A}-\lambda_{A\to T}-\lambda_{A\to S}+\lambda_{F\to A}(t),\\ {\dot{\xi }}_{T} & = & \lambda_{A\to T}-\lambda_{T\to A}\\ {\dot{\xi }}_{S} & = & \lambda_{A\to S}-\lambda_{S\to A}-\lambda_{S\to D}+\lambda_{D\to S} \end{array}\right.$$ (10)

As shown in (10) the right–hand side of (9) depends on *t* and also on the state matrix $\xi (t)={\left(\begin{array}{c} \xi_{A}(t)\\ \xi_{T}(t)\\ \xi_{S}(t) \end{array}\right)}^{T}$. In other words, fluxes between compartments are nonlinearly dependent upon the contents of those compartments. Here are the results of Eq (1), assuming 𝔹=𝔹(ℂ,t) is a state– and time–dependent compartmental matrix:

$$\left[\begin{array}{ccc} -\xi_{A}^{-1}\left(\lambda_{A\to T}+\lambda_{A\to S}\right) & \xi_{T}^{-1}\lambda_{T\to A} & \xi_{S}^{-1}\lambda_{S\to A}\\ \xi_{A}^{-1}\lambda_{A\to T} & -\xi_{T}^{-1}\lambda_{T\to A} & 0\\ \xi_{A}^{-1}\lambda_{A\to S} & 0 & -\xi_{S}^{-1}\left(\lambda_{S\to A}+\lambda_{S\to D}\right) \end{array}\right]$$ (11)

and $m(t):={\left(\lambda_{F\to A}(t),0,\lambda_{D\to S}\right)}^{T}$ , and then the plant fits into (1) without considering temperature. From the global climate carbon emissions theory, the authors presents the new fuzzy rules in Table 1. To describe the nature A compartmental nonlinear time–dependent system can be stated as follows:

$$\left\{ \begin{array}{ccc} \dot{\xi }(t) & = & 𝔹(\mathbb{C},t)\xi (t)+m(t),\;t>t_{0},\\ \xi \left(t_{0}\right) & = & \xi_{0} \end{array}\right.$$ (12)

From the equilibrium point of the uncontrolled system (u(t)=1), we ran it at the point $\xi_{0}={\left(700,3000,1000\right)}^{T}$ from 1765 forward until 1900, at which point the control function was turned on, u(t)=ψ(κ(t)). The global carbon cycle was assumed to be controlled by humanity after 1900, as all fossil fuel emissions were allowed until this date.

**Fig 12:**
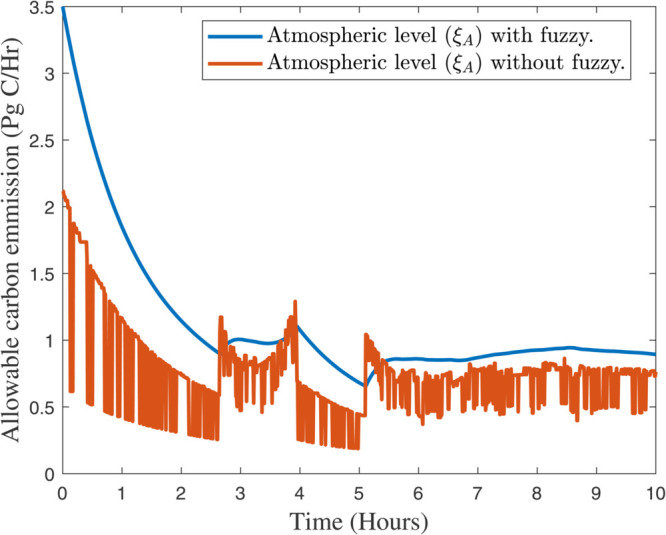
Response of atmospheric ($\xi_{A}$) with and without fuzzy rules.

**Fig 13:**
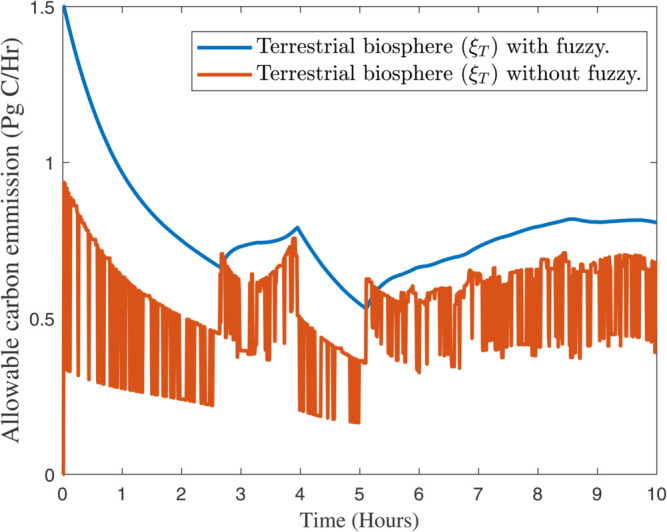
Response of terrestrial biosphere ($\xi_{T}$) with and without fuzzy rules.

**Fig 14:**
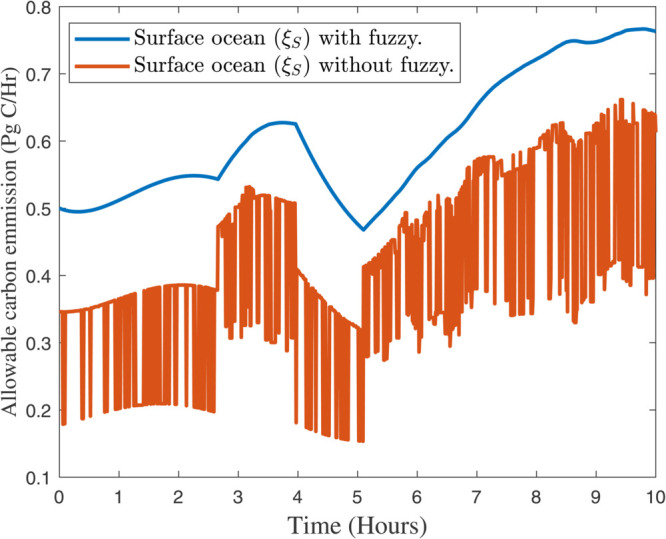
Response of Surface ocean ($\xi_{S}$) with and without fuzzy rules.

After selecting the fuzzy rules from the Sect 4, the effectiveness of our proposed algorithms can be found in Figs 12, 13, and 14 respectively. In Fig 12, the response of atmospheric ($\xi_{A}$) with and without fuzzy rules can be analyzed. While in Figs 13 and 14 presented the response of terrestrial biosphere ($\xi_{T}$) and surface ocean ($\xi_{S}$) with and without fuzzy rules. Fuzzy logic and fuzzy rules play a significant role in modeling and understanding complex systems like global climate. Here are the key aspects of how fuzzy rules contribute to global climate studies, e.g., *Handling Uncertainty and Ambiguity*, *Modeling Complex Relationships*, *Integrating Diverse Data Sources*, *Improving Climate Prediction Models*, *Adaptive Systems, and Risk Assessment* and *Decision Support*. However, there are some practical examples that can be effective by using our new proposed algorithm in *Climate Change Impact Studies*, *Weather Forecasting*, and *Environmental Monitoring*. In summary, fuzzy rules are invaluable in global climate studies for their ability to handle complexity, integrate diverse data, and improve predictive models, ultimately aiding in more effective climate management and decision–making.

### 6.1 Prediction analysis of external/internal air temperature

**Fig 15:**
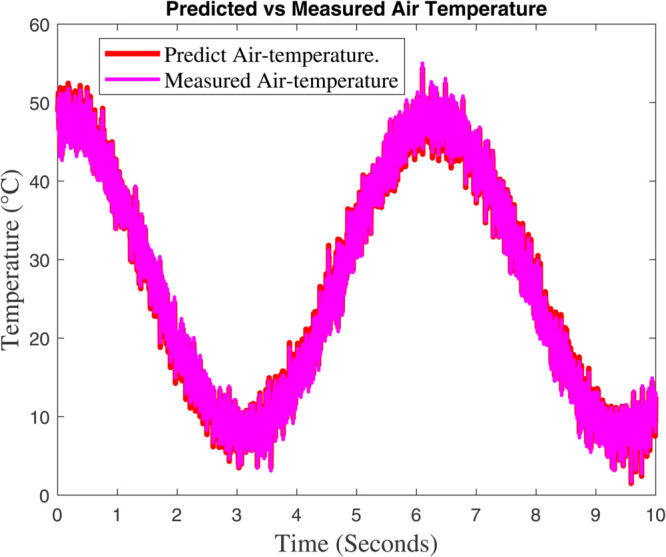
Greenhouse’s interior temperature and the fuzzy model’s prediction.

**Fig 16:**
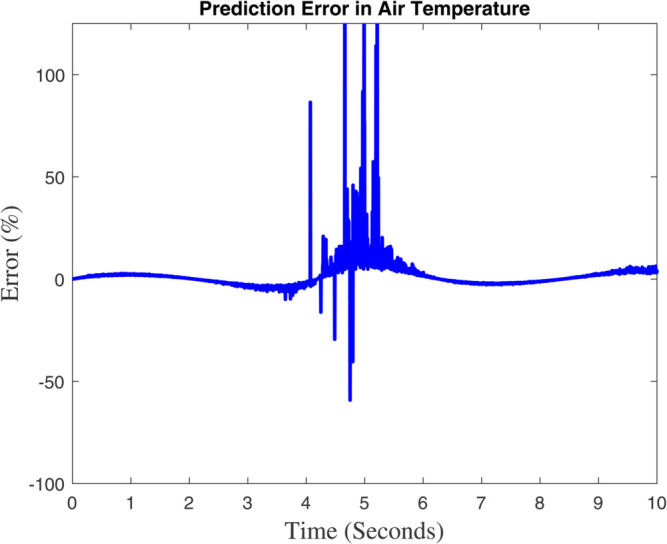
The Global climate model’s outcome for internal temperature differ.

Two figures are showing the predicted vs. measured air temperature data and the associated prediction error are included in Figs 15 and 16. The fact that the two lines closely follow one another suggests that the model’s prediction and the actual temperature are in good agreement. A periodic temperature variation that may be impacted by external or control system elements is suggested by the data’s apparent sinusoidal rhythm. The percentage discrepancy between the measured and predicted temperatures is displayed in this graphic. There is a noticeable increase in the error between 4 and 6 seconds, even though it stays minor and steady for the most of the time–line. This implies that the model had trouble correctly predicting the temperature during this time, possibly as a result of abrupt changes in the surrounding environment or quick variations. The tight match between the anticipated and measured data in Fig 15 shows how well the model predicts temperature. The steep error spikes in Fig 6 imply that either there was noise in the data during that time period or the model would have trouble capturing abrupt temperature changes. These might be caused by inaccurate sensors, abrupt changes in the environment, or restrictions in the model’s capacity to make predictions. Although the model works well in general, it might need to be improved in order to lessen the error spikes that are shown at specific times. These disparities might be reduced with the use of strategies like better data filtering, better model tuning, or more feature inclusion.

**Fig 17:**
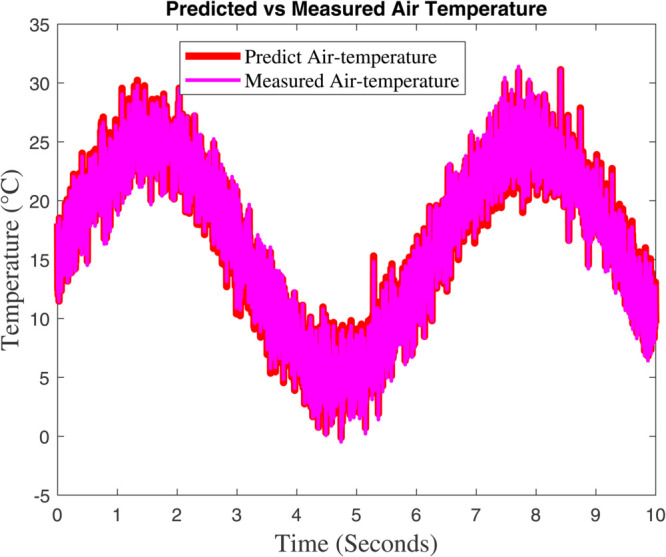
Greenhouse’s exterior temperature and the dynamic model’s prediction.

**Fig 18:**
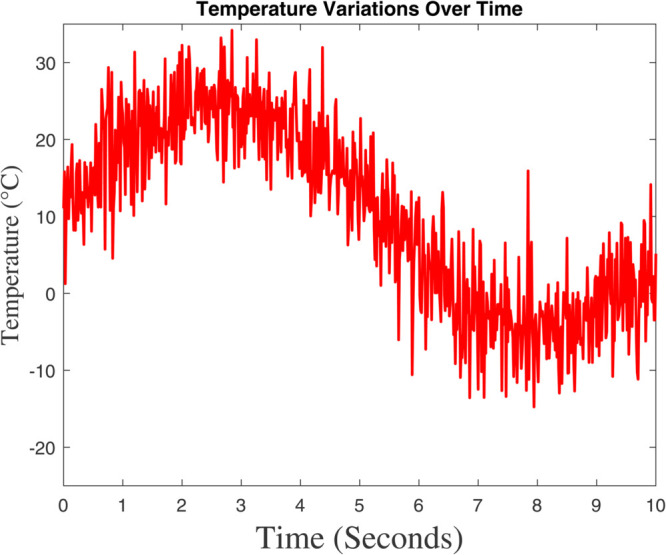
The Global climate model’s outcome for internal temperature differ.

Fig 15 shows that the dynamic model accurately forecasts the temperature outside the greenhouse. The data exhibits a sinusoidal pattern, indicating recurring variations that are probably impacted by environmental cycles like exposure to sunlight, meteorological circumstances, or temperature regulation systems. There are slight variations between the expected and actual temperatures, especially during the peak and trough periods, which could indicate model flaws or outside disturbances. The temperature trend shows greater variability during periods of peak temperature, suggesting increased sensitivity to external factors like sunlight intensity, wind patterns, or sensor noise. It also generally corresponds with the sinusoidal pattern seen in Fig 15, indicating periodic environmental changes. These variations’ fluctuating amplitudes reflect changing environmental conditions. In Figs 17 and 18 show a significant agreement between the predicted and observed values, indicating that the dynamic model accurately and minimally represents the overall temperature trend. In Fig 17’s temperature variability demonstrates how intricate and dynamic the greenhouse’s external environment is. Although the model seems stable, it might be improved to better manage abrupt changes (seen as noise in Fig 18) in order to increase prediction accuracy. The dynamic model exhibits a significant association with measured data and accurately forecasts the greenhouse’s outside temperature. Sharp temperature swings, however, imply that the model’s performance might be further enhanced by addressing environmental variability or reducing noise.

### 6.2 The control cost function u(t)=ψ(z(t))

**Fig 19:**
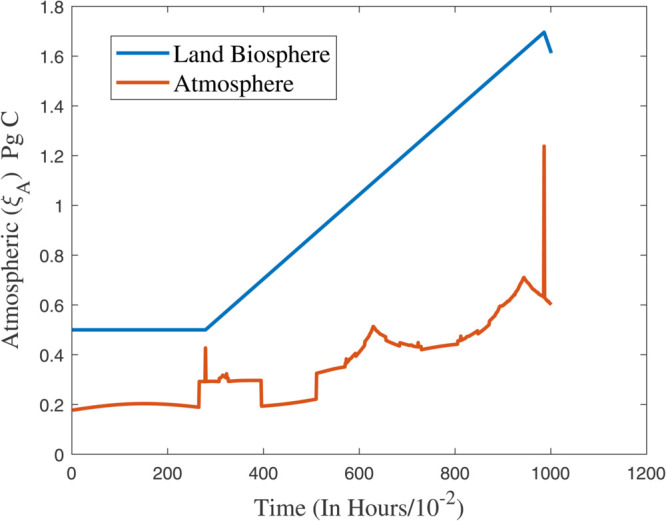
Response of atmospheric ($\xi_{A}$) in the atmosphere, and land biosphere.

**Fig 20:**
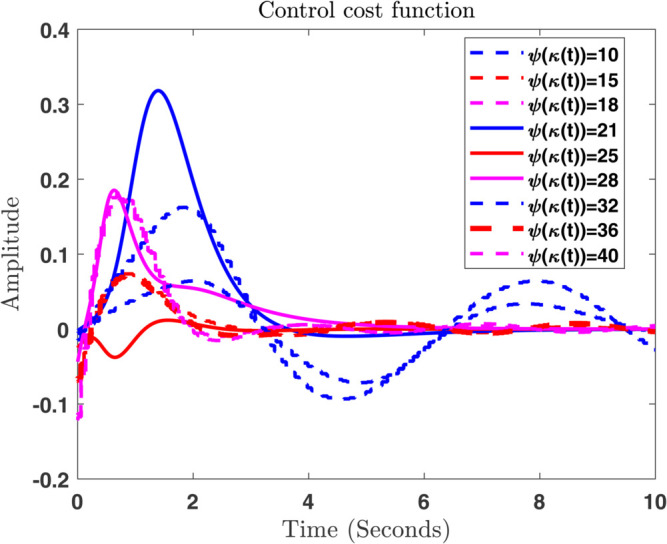
Convergence of the control inputs at different values of ψ(κ(t)).

As a control objective, [36] proposes a closed–loop controller that matches the fossil fuel demand to the maximum extent possible while preventing compartmental network overflows. If there is only one output, then the formula is u(t)=ψ(κ(t)) , where *ψ* is as follows: $\psi :{ℛ}_{+}\mapsto {ℛ}_{+}$. No restriction is placed on the notation, throughout the time as it is continuously differentiable and rising gradually, and fulfils the conditions ψ(0)=0 as well as $\lim_{\kappa \to \infty }\psi (\kappa )=1$. Using this freedom, the controller could be improved, e.g., by parameterizing *ψ* with many tunable parameters. Instead of aiming for maximal simplicity, we chose to represent the imaginary buffer with $\kappa_{0}=\kappa_{max}$, its initial value. A smooth controller installation was also important to us. As a result, $\kappa \geq \kappa_{max}$ is equal to ψ(κ)=1 . During the time when the buffer is empty and the storage κ is full, the controller is inactive, but once the buffer competes and the rest of the storage κ decreases, the controller becomes active. In Fig 19, the readers can observed the response of atmospheric ($\xi_{A}$) in the atmosphere, and land biosphere. In addition, the convergence of the control inputs at different values of ψ(κ(t)) can be observed in Fig 20.

Using the mathematical form given above, we can also write the control function in the following way:


$$\psi (\kappa )=\left\{ \begin{array}{cc} g & \kappa \leq \kappa_{\max},\\ 1 & \kappa >\kappa_{\max}, \end{array}\right.$$

In accordance of *g* with the following requirements:


$$\left\{ \begin{array}{c} g(0)=0,\\ g\left(\kappa_{\max}\right)=1,\\ g^{\prime }(0)=1/\kappa_{\max},\\ g^{\prime }\left(\kappa_{\max}\right)=0, \end{array}\right.$$

The coefficients are uniquely determined when a cubic Hermite spline is used to solve the cubic equation.


$$g(\kappa )=\alpha_{0}+\alpha_{1}\kappa_{1}+\alpha_{2}\kappa^{2}+\alpha_{3}\kappa^{3}.$$

Consequently, the controller’s timing is determined only by $\kappa_{\max}$ , which determines when it turns off and at what speed it operates. Controllers with steep slopes can react quickly at low values of $\kappa_{max}$, while those with steep slopes at higher values of $\kappa_{max}$ react slowly. In contrast to original model, we assume that the *air–ocean flow* ($\lambda_{A\to S}$) and the *air–biosphere flow* ($\lambda_{A\to T}$) concentrate at 200 and 90 *PgC* year ^−1^, respectively. The idea that natural sinks are saturated is consistent with previous studies. Controlling fossil fuel emissions can avoid this congestion by allowing emissions to be controlled based on a known demand *p*. By allowing only a small percentage *ψ* of the demand *p*, the authors implement an output feedback control scheme. The auxiliary variable κ is defined as follows:

$$\dot{\psi }(t)=y(t)-\psi (\kappa )).p(t)$$ (13)

where


$$y(t)=\lambda_{S\to D}-\lambda_{D\to S}$$

*Carbon flux* to the deep ocean is the amount of carbon that leaves the system. *y*(*t*) is a measure of the net outflow from the system, which feeds the variable κ. After that, drain by a controlled input whose size is based on the virtual pool’s content via a monotonic and continuous–time differentiable at first input. A function with no constraints *ψ* is on the other hand. This arrangement prevents congestion *ψ* as it maintains the total mass constant while generating a physically closed virtual system. Congestion control can be used as an additional goal in a realistic scenario based on the freedom to choose. In terms of some desired properties, it may be optimized. Since no assumption of optimality is necessary for this example, such optimization is obviously outside its purview.

**Fig 21:**
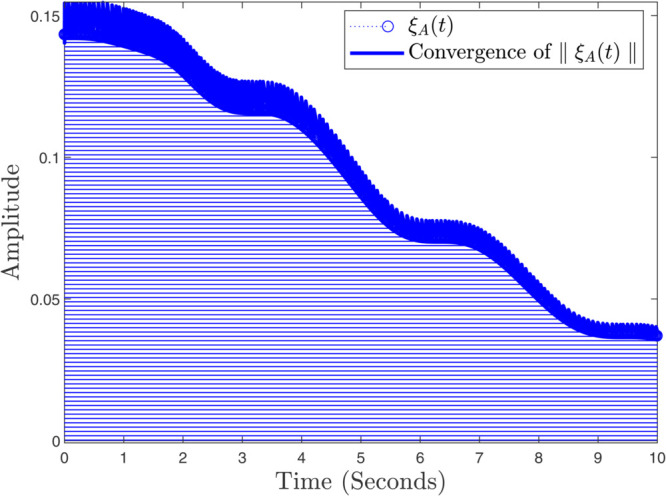
Convergence ratio of $\xi_{A}(t)$ and $∥\xi_{A}(t)∥$.

**Fig 22:**
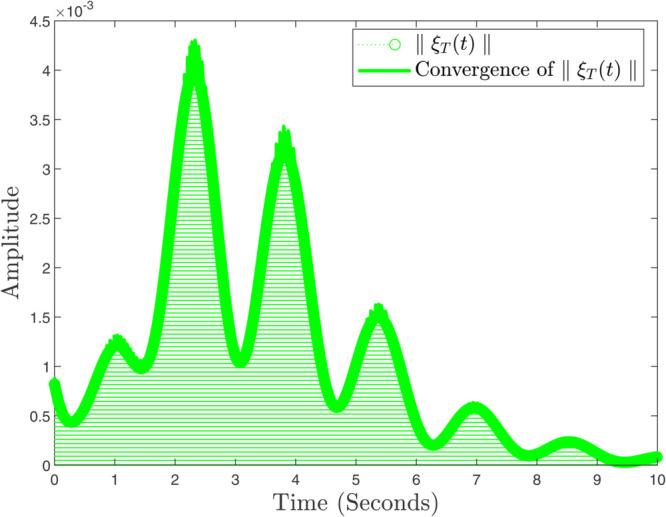
Convergence ratio of $\xi_{T}(t)$ and $∥\xi_{T}(t)∥$.

**Fig 23:**
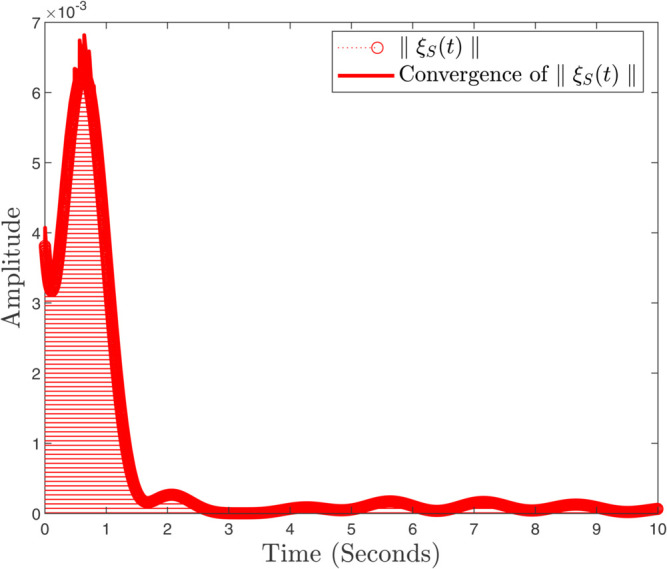
Convergence ratio of $\xi_{S}(t)$ and $∥\xi_{S}(t)∥$.

**Fig 24:**
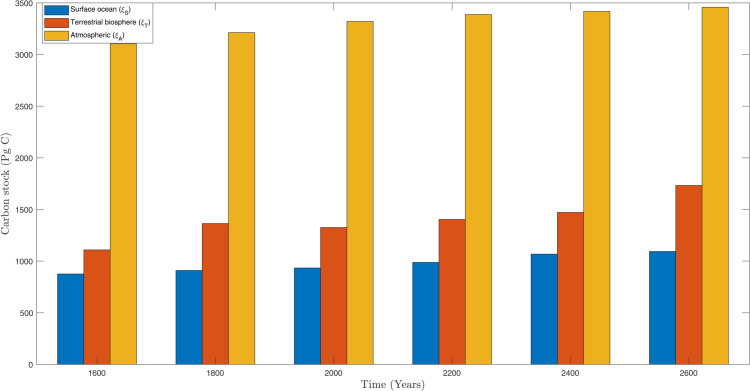
The influence of varying $\kappa_{max}$ values on the regulatory framework and its efficacy in preventing the buildup of atmospheric carbon within the context of thermal energy storage.

In instances where $\kappa_{max}$ assumes minimal values, the controller exhibits a pronounced gradient, indicating its capacity for rapid response; conversely, as the parameter attains greater magnitudes, the controller demonstrates a more gradual reaction. In the same way, the convergence ratio of all trajectories has been shown in Figs 21, 22, and 23. The influence of varying $\kappa_{max}$ values on the regulatory framework and its efficacy in preventing the buildup of atmospheric carbon within the context of thermal energy storage can be observed in Fig 24.

**Fig 25:**
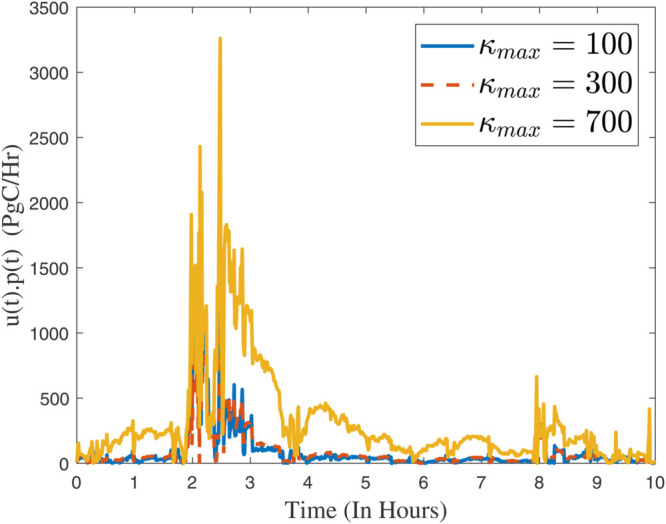
The quantity of carbon emissions permitted annually, measured in PgC, as determined by $\lambda_{F\to A}(t)=u(t).p(t)$.

The quantity of carbon emissions permitted annually, measured in *PgC*, as determined by $\lambda_{F\to A}(t)=u(t).p(t)$. The *terrestrial biosphere* refers to the portion of *Earth’s surface* that supports life, encompassing all ecosystems on land. It includes forests, grasslands, deserts, tundra, and other terrestrial ecosystems. An essential component of the global carbon cycle is the terrestrial biosphere, influencing climate regulation, biodiversity, and the provision of ecosystem services such as food, water, and raw materials. From the Fig 25, for low $\kappa_{max}$ values, the controller exhibits a sharp incline, enabling rapid response, whereas for higher parameter values, the controller’s reaction is more gradual.

**Remark 4.** *The outputs from y(t) the carbon–climate system are utilized in various ways to inform control strategies and policy decisions. Here are the key areas where these outputs are applied for control Input for Feedback Mechanisms; System Identification and Observability, and Policy Evaluation and Adjustment. The outputs from the system, such as measurements of atmospheric *CO**_*2*_ *concentrations and surface temperatures, are crucial for closed–loop control systems. These outputs help in adjusting control actions based on real–time data, allowing for adaptive management of the carbon–climate system. Model identification, which entails building mathematical models from observed data, depends on outputs. Understanding the dynamics of the carbon–climate system and evaluating its observability–the capacity to deduce the system’s state from sparse observations are aided by this approach. Monitoring plant’s outputs offer vital data for assessing how well–functioning the policies in place are.*

Data sharing is applicable to this article as no data–sets were generated or analyzed during the current study. In case of ethical statement, both human and/or animal investigations were not approved for this research.

## 7 Conclusions

It is possible to model *Carbon–climate control* as a closed–loop issue that continuously modifies the system’s activity in response to measurements and uncertainty. In this paper, we provide a new mathematical description of the *carbon–climate structure* based on fuzzy systems in order to formalize the closed–loop control problem. A compartmentalized representation of the *global carbon structure* can be used to achieve particular mathematical properties such as dynamic stability by utilizing the compartmental structure of the system of equations. With our simulation results, the authors demonstrated that fuel requirement and the ability of the plant to transport *geological reservoirs* for *carbon*, such as the deep sea, could be effectively managed to manage the global carbon cycle. By analyzing the demand and internal dynamics of the system, we obtained a mathematical rule for prescribing actions. This rule can be applied to adaptive carbon cycle management. Unlike scenario–based emission route prescriptions, this technique could be useful for policy decisions made on shorter time horizons than those imposed by long–term scenarios.

### 7.1 Future direction

Fuzzy logic is used to model and control the intricacies and uncertainties in climate systems in order to control the global climate based on fuzzy systems with carbon emissions. The complexity and uncertainty of climate models, data quality and availability, scalability issues, interpretability and transparency, integration with other models, and adaptability and evolution are some of the limitations of this approach, despite its potential benefits. Fuzzy systems are a useful tool for handling the uncertainties and complexity of climate modeling, but they have a lot of drawbacks. To overcome these constraints, fuzzy logic must be combined with other modeling techniques, data quality must be improved, and computer power must be increased. Collaboration between scientists and policymakers is necessary to create resilient, open, and flexible systems that can efficiently guide climate action and *decision–making*. Furthermore, our recently proposed fuzzy approach [39] can be used to improve a few more directions. Additionally, the following *T–S models* can be used to further improve the efficiency of *Carbon–climate control*:

- *Hybrid Approaches*: Consider merging state–of–the–art machine learning techniques with *T–S models* to increase model adaptability and forecast accuracy.
- *Localized Adaptation*: Considering the need for localized *T–S models* that account for regional climate variations, guaranteeing precise forecasts appropriate for specific locations.
- *Uncertainty Management*: Developing trustworthy techniques to measure and control uncertainty in *T–S models* in order to generate forecasts that are more precise and comprehensible.
- *Long–Term Modeling*: Research techniques to improve the *T–S model’s* ability to project the climate over the long run while accounting for the dynamic nature of climate systems.
- *Socio–Economic Consideration*: We can bridge the gap between social dynamics and climate science by incorporating socio-economic factors, human behavior, and policy consequences into *T–S models*.
- *Interdisciplinary Collaboration*: Fostering communication and collaboration between climate scientists, mathematicians, decision-makers, and other experts to manage the complex relationships and challenges associated with the application of the *T–S model* in climate change modeling.

The *Takagi–Sugeno Fuzzy Models (T–S models)* offer a promising approach to understanding climate change dynamics, they are not without challenges. These concerns need to be recognized and addressed in order to ensure that *T–S models* are used in climate change research in an accurate and significant manner. In Data inaccuracy, high–quality climate data is necessary for accurate forecasts, but that data gaps and uncertainties can inject mistakes into the models, reducing the reliability of the results. Similarly, model complexity for the *T–S models* may involve additional variables and rules due to the complexity of climate systems, which could impact their accessibility and computational efficacy. For the model complexity, the *T–S models* may involve more factors and rules due to the complexity of climate systems, which could impact their accessibility and computational efficacy.
